# ­­­­­­Topical Antifungal Prescribing for Medicare Part D Beneficiaries — United States, 2021

**DOI:** 10.15585/mmwr.mm7301a1

**Published:** 2024-01-11

**Authors:** Kaitlin Benedict, Dallas J. Smith, Tom Chiller, Shari R. Lipner, Jeremy A. W. Gold

**Affiliations:** ^1^Division of Foodborne, Waterborne, and Environmental Diseases, National Center for Emerging and Zoonotic Infectious Diseases, CDC; ^2^Israel Englander Department of Dermatology, Weill Cornell Medicine, New York, New York.

**Summary**

**What is already known about this topic?**

Severe antimicrobial-resistant superficial fungal infections have recently been detected in the United States; evaluating topical antifungal use is an initial step in developing strategies to prevent the global emergence and spread of these infections.

**What is added by this report?**

A total of 6.5 million topical antifungal prescriptions, costing $231 million, were filled for Medicare Part D beneficiaries in 2021, approximately one prescription for every eight beneficiaries. Most prescriptions were written by primary care physicians, nurse practitioners, or physician assistants.

**What are the implications for public health practice?**

The large volume of topical antifungal prescriptions in the context of emerging resistance highlights the need to better understand current prescribing practices and to encourage judicious prescribing by clinicians and improve patient education about recommended use.

## Abstract

Incorrect use of topical antifungals and antifungal-corticosteroid combinations is likely contributing to the global emergence and spread of severe antimicrobial-resistant superficial fungal infections, which have recently been detected in the United States. Understanding prescribing patterns is an initial step in establishing and promoting recommended use of these medications. Using 2021 Medicare Part D data, CDC examined prescription volumes, rates, and costs for topical antifungals (including topical combination antifungal-corticosteroid medications). Total prescription volumes were compared between higher-volume prescribers (top 10% of topical antifungal prescribers by volume) and lower-volume prescribers. During 2021, approximately 6.5 million topical antifungal prescriptions were filled (134 prescriptions per 1,000 beneficiaries), at a total cost of $231 million. Among 1,017,417 unique prescribers, 130,637 (12.8%) prescribed topical antifungals. Primary care physicians wrote the highest percentage of prescriptions (40.0%), followed by nurse practitioners or physician assistants (21.4%), dermatologists (17.6%), and podiatrists (14.1%). Higher-volume prescribers wrote 44.2% (2.9 million) of all prescriptions. This study found that enough topical antifungal prescriptions were written for approximately one of every eight Medicare Part D beneficiaries in 2021, and 10% of antifungal prescribers prescribed nearly one half of these medications. In the setting of emerging antimicrobial resistance, these findings highlight the importance of expanding efforts to understand current prescribing practices while encouraging judicious prescribing by clinicians and providing patient education about proper use.

## Introduction

Superficial fungal skin infections have an estimated lifetime prevalence of more than 20% worldwide and are particularly common among adults aged ≥65 years (*1*–*3*). The emergence and spread of antimicrobial-resistant superficial fungal infections, especially dermatophytosis (also known as ringworm or tinea), has led to large outbreaks of extensive, recalcitrant skin infections in South Asia that frequently do not respond to topical antifungals or first-line oral therapies. This emergence and spread are likely exacerbated by the overuse and misuse of topical antifungals, particularly antifungal-corticosteroid combination creams (*1*,*4*). Cases of antimicrobial-resistant dermatophytosis have been identified in at least 11 U.S. states (*5*), with patients experiencing extensive lesions and delays in diagnosis (*6*). In the United States, nonrecommended topical antifungal prescribing is likely common, because diagnosis of cutaneous fungal infections by visual inspection is frequently incorrect, including among board-certified dermatologists (*7*), and clinicians across specialties rarely perform confirmatory diagnostic testing (*2*,*8*). Understanding prescribing patterns, including identification of clinicians who prescribe a disproportionate volume of topical antifungals, might help establish and promote correct use of these medications (*9*). Centers for Medicare & Medicaid Services (CMS) data were used to characterize prescribing volume of topical antifungal medications among Medicare Part D beneficiaries in the United States during 2021.

## Methods

### Data Source

Approximately 48.8 million (76%) Medicare beneficiaries were enrolled in the Part D prescription drug benefit program in 2021,*^,^^†^ the most recent year of data available for this study. The publicly available CMS Medicare Part D Prescribers–by Provider and Drug data set^§^ contains information on the total number of prescriptions (including refills) and total drug costs,^¶^ aggregated by National Provider Identifier number and drug name. The data set excludes clinician records with <11 prescriptions. Prescribers from outside the United States or whose U.S. Census Bureau region was unknown were excluded from the analysis.** The Medicare Monthly Enrollment dataset^††^ was used to ascertain the total number of Medicare Part D beneficiaries.

### Data Analysis

Prescription volume (i.e., number of prescriptions), total costs, and average costs for topical antifungal and topical antifungal-corticosteroid drugs covered by Medicare Part D^§§^ were assessed. Prescription rates per 1,000 beneficiaries were calculated by drug and region and to calculate prescription rates per 1,000 beneficiaries by drug and region. Prescription rates per prescriber were calculated by tabulating number of providers overall and by provider type (primary care physician [internal medicine or family medicine physician], nurse practitioner or physician assistant, dermatologist, podiatrist, and other) in the Medicare Part D Prescribers–by Provider and Drug data set as the denominator.

Higher-volume prescribers were defined as those within the top 10th percentile of prescriber-level topical antifungal prescriptions by volume. The volume and percentage of topical antifungal prescriptions written by higher-volume prescribers compared with lower-volume prescribers (bottom 90th percentile) were assessed overall and by prescriber type. Analyses were conducted using SAS software (version 9.4; SAS Institute). This activity was reviewed by CDC, deemed not research, and was conducted consistent with applicable federal law and CDC policy.^¶¶^

## Results

### Prescriptions

During 2021, a total of 6.5 million topical antifungal prescriptions were filled by Part D beneficiaries (overall rate = 134.0 prescriptions per 1,000 beneficiaries) (Table 1). By volume, the most common prescriptions were for ketoconazole (2.4 million [36.6%]), nystatin (1.9 million [29.0%]), and clotrimazole-betamethasone dipropionate (0.9 million [14.7%]). The total cost for all topical antifungal prescriptions was $231 million. The highest average per prescription costs were for efinaconazole ($1,035.38), tavaborole ($784.63), and oxiconazole ($729.84); the lowest average costs were for nystatin ($25.66), clotrimazole-betamethasone dipropionate ($27.82), clotrimazole ($30.36), and ketoconazole ($30.69). By U.S. Census Bureau region, the highest prescription rate was in the Northeast (188.0 prescriptions per 1,000 beneficiaries), followed by the South (138.1 per 1,000).

**TABLE 1:** Number of topical antifungal prescriptions, prescriptions per 1,000 beneficiaries, and cost, by drug and U.S. Census Bureau region — United States, 2021

Characteristic	No. of prescriptions (% of all prescriptions)	Prescriptions per 1,000 beneficiaries	Aggregate cost, all prescriptions, USD*	Avg. cost per prescription, USD*
**Antifungal drug**				
Ketoconazole^†^	2,364,169 (36.6)	49.1	72,556,081.61	30.69
Nystatin	1,871,368 (29.0)	38.9	48,025,095.10	25.66
Clotrimazole-betamethasone dipropionate	945,838 (14.7)	19.6	26,311,901.12	27.82
Ciclopirox	657,986 (10.2)	13.7	22,739,088.92	34.56
Clotrimazole	397,603 (6.2)	8.3	12,070,066.47	30.36
Econazole	75,675 (1.2)	1.6	4,414,180.46	58.33
Nystatin-triamcinolone	55,276 (0.9)	1.1	2,928,976.33	52.99
Terconazole	32,203 (0.5)	0.7	1,051,091.05	32.64
Efinaconazole	17,881 (0.3)	0.4	18,513,585.90	1,035.38
Oxiconazole	14,892 (0.2)	0.3	10,868,838.98	729.84
Naftifine	13,532 (0.2)	0.3	5,018,673.14	370.87
Tavaborole	8,317 (0.1)	0.2	6,525,735.61	784.63
Other^§^	400 (—)	—	185,439.42	463.60
**U.S. Census Bureau region**				
Northeast	1,677,727 (26)	188.0	78,235,437.54	46.63
Midwest	1,198,093 (19)	112.2	34,204,692.89	28.55
South	2,489,321 (39)	138.1	74,554,713.01	29.95
West	1,089,999 (17)	103.5	44,213,910.67	40.56
**Total**	**6,455,140 (100)**	**134.0**	**231,208,754.11**	**35.82**

* All costs are in U.S. dollars.

^†^ It was not possible to distinguish oral from topical ketoconazole, but ketoconazole was included in the analysis because oral ketoconazole use is discouraged because of safety concerns. https://www.fda.gov/drugs/drug-safety-and-availability/fda-drug-safety-communication-fda-warns-prescribing-nizoral-ketoconazole-oral-tablets-unapproved

^§^ Butenafine (41 prescriptions), luliconazole (169), miconazole (155), and sertaconazole (35).

### Prescribers

Among 1,017,417 unique prescribers, 130,637 (12.8%) prescribed topical antifungals (Table 2). The number of prescriptions per provider was highest for dermatologists (87.1), followed by podiatrists (67.2), and primary care physicians (12.3). Among 6.5 million topical antifungal prescriptions, the most were written by primary care physicians (2.6 million [40.0%], followed by nurse practitioners or physician assistants (1.4 million [21.4%]), dermatologists (1.1 million [17.6%]), and podiatrists (0.9 million [14.1%]). Among all topical antifungal prescriptions, 44.2% (2.9 million) were written by the top 10% (13,106) of topical antifungal prescribers. By provider type, the percentage of topical antifungal prescriptions written by higher-volume topical antifungal prescribers ranged from 35.5% for dermatologists to 54.8% for podiatrists.

**TABLE 2:** Number of topical antifungal prescriptions per provider and prescribing volume among higher-volume* and lower-volume^†^ prescribers, by provider type for Medicare Part D beneficiaries — United States, 2021

Provider type (no.)	No. of prescriptions per provider	All topical antifungal prescribers		Higher-volume prescribers (top 10%)		Lower-volume prescribers (bottom 90%)	
		No. of prescribers	No. of prescriptions (% of total)^§^	No. of prescribers	No. of prescriptions (% of total)^¶^	No. of prescribers	No. of prescriptions (% of total)^¶^
Primary care physician (209,169)	12.3	61,735	2,579,045 (40.0)	6,200	973,824 (37.8)	55,535	1,605,221 (62.2)
NP or PA (263,999)	5.2	34,476	1,379,981 (21.4)	3,428	570,577 (41.3)	31,048	809,404 (58.7)
Dermatologist (13,029)	87.1	10,735	1,134,347 (17.6)	1,068	402,750 (35.5)	9,667	731,597 (64.5)
Podiatrist (13,527)	67.2	8,401	909,569 (14.1)	838	498,210 (54.8)	7,563	411,359 (45.2)
Other (517,693)	0.9	15,290	452,198 (7.0)	1,507	165,742 (36.7)	13,783	286,456 (63.3)
**Total (1,017,417)**	**6.3**	**130,637**	**6,455,140 (100.0)**	**13,106**	**2,851,394 (44.2)**	**117,531**	**3,603,746 (55.8)**

**Abbreviations:** NP = nurse practitioner; PA = physician assistant.

* Top 10% of topical antifungal providers, by volume.

^†^ Bottom 90% of topical antifungal providers, by volume.

^§^ Column percentage.

^¶^ Row percentage.

## Discussion

This analysis of publicly available CMS data found that 6.5 million topical antifungal prescriptions (enough to provide one prescription to more than one eighth of all beneficiaries) were written for Medicare Part D beneficiaries in 2021, at a cost of $231 million. The actual volume of topical antifungal use among the study population is likely considerably higher than that identified in this study because most topical antifungals can be purchased over the counter without a prescription; such topical antifungal use is not recorded in CMS data and is an important consideration for potential antifungal stewardship efforts. The large volume of topical antifungals used in the United States warrants increased attention given the infrequent use of confirmatory testing, inaccuracy of diagnosis made by physical examination alone, and the recent emergence of severe and antimicrobial-resistant superficial skin infections (*1*,*5*,*6*). To help control the emergence and spread of antimicrobial-resistant superficial fungal infections and help promote the appropriateness of topical antifungal prescribing, health care providers could use diagnostic testing*** whenever possible to confirm suspected superficial fungal infections. Further, health care providers can educate patients about prognosis, benefits, and harms of topical antifungal and combination antifungal-corticosteroid treatment (both prescription and over- the-counter), and the importance of using these medications as prescribed or according to manufacturer instructions.

### Variation in Prescribing by Prescriber Type and Region

The largest number of topical antifungal prescriptions was written by primary care physicians, nurse practitioners, or physician assistants, suggesting that efforts to determine and improve appropriateness of prescribing could prioritize these groups. Although dermatologists and podiatrists had lower prescribing volumes compared with other groups, they had higher per-provider prescribing rates. This observation could reflect that dermatologists and podiatrists might see patients with superficial fungal infections more frequently than do other provider types. In contrast to systemic antibiotic prescribing, which is highest in the South (*9*), topical antifungal prescribing rates were highest in the Northeast. Reasons for this finding are unclear but could reflect a higher prevalence of superficial fungal infections, more ready access to medical care, or less frequent use of over-the-counter topical antifungal medications in the Northeast compared with that in other regions.

### High Volume Prescribers and Prescriptions

As with antibiotic prescribing for Medicare Part D beneficiaries, 10% of prescribers wrote a disproportionately large percentage (>40%) of topical antifungal prescriptions (*9*). Among podiatrists, the top 10% of prescribers wrote more than one half of topical antifungal prescriptions. These findings suggest potential opportunities to prioritize higher-volume topical antifungal prescribers for antimicrobial stewardship interventions using evidence-based techniques such as peer comparison audit and feedback; however, additional data are needed to determine whether topical antifungal prescribing rates correlate with rates of incorrect prescribing, as shown for systemic antibiotics in primary care settings (*9*).

The large volume of clotrimazole-betamethasone dipropionate prescriptions (0.9 million; 15% of all topical antifungal prescriptions) is potentially concerning, as use of combination topical medications containing corticosteroids and antifungal agents has been proposed as a potential driver of emerging antimicrobial-resistant dermatophytosis (*10*). In addition, clotrimazole-betamethasone dipropionate contains a high-potency steroid that can cause skin damage if applied to intertriginous areas as well as hypothalamic‐pituitary‐adrenal axis suppression if used for a prolonged time or over a large body surface area.^†††^ Clinicians should be aware of the potential risks associated with clotrimazole-betamethasone dipropionate use and consider alternatives such as antifungal monotherapy, with a short course of low-potency corticosteroid treatment added if needed for symptoms such as severe pruritis.^§§§^

### Limitations

The findings in this report are subject to at least four limitations. First, the data set does not contain information on individual patients, drug indication (i.e., candidiasis versus dermatophytosis), or diagnostic testing, so prescribing appropriateness could not be determined. Second, the data set analyzed only identifies prescriptions for Medicare Part D beneficiaries and therefore does not represent all Medicare beneficiaries; topical antifungal prescribing patterns might differ among other populations. Third, the data set only contained information on prescription topical antifungals and did not capture over-the-counter topical antifungal use; therefore, actual topical antifungal use is likely underestimated. Finally, this study likely underestimates the total volume of topical antifungal drug prescribing among Medicare Part D beneficiaries because records for some lower-volume prescribers (those with <11 prescriptions per year for any given drug) are not included in the data set, and prescribers whose census region was unknown were excluded.

### Implications for Public Health Practice

The substantial volume of topical antifungal and antifungal-corticosteroid prescriptions among Medicare Part D beneficiaries in the setting of emerging resistant infections underscores the need to evaluate current practices of topical antifungal use. Health care providers should be judicious in prescribing topical antifungals and combination antifungal-corticosteroid medications for suspected superficial fungal infections, using testing when feasible to confirm diagnoses, and can educate patients about the correct use of topical antifungals and combination antifungal-corticosteroids.

## Footnotes

* Medicare, the federal health insurance program, is available to most persons aged ≥65 years, certain persons aged <65 years who are receiving disability benefits, and persons with end stage renal disease. Medicare Part D is the part of Medicare that helps cover the cost of prescription drugs. https://www.medicare.gov/what-medicare-covers/your-medicare-coverage-choices/whats-medicare

†
https://data.cms.gov/summary-statistics-on-beneficiary-enrollment/medicare-and-medicaid-reports/cms-program-statistics-medicare-part-d-enrollment

§
https://data.cms.gov/provider-summary-by-type-of-service/medicare-part-d-prescribers/medicare-part-d-prescribers-by-provider-and-drug

¶ Cost includes the ingredient cost, dispensing fee, and sales tax and is based on the amounts paid by the Part D plan, Medicare beneficiary, government subsidies, and any other third-party payers. https://data.cms.gov/resources/medicare-part-d-prescribers-by-provider-and-drug-data-dictionary

**
https://www2.census.gov/geo/pdfs/maps-data/maps/reference/us_regdiv.pdf

††
https://data.cms.gov/summary-statistics-on-beneficiary-enrollment/medicare-and-medicaid-reports/medicare-monthly-enrollment

§§ Butenafine, butoconazole, ciclopirox, clotrimazole, clotrimazole-betamethasone dipropionate, econazole, efinaconazole, ketoconazole, luliconazole, miconazole, naftifine, nystatin, nystatin-triamcinalone, oxiconazole, sertaconazole, sulconazole, tavaborole, and terconazole.

¶¶ 45 C.F.R. part 46.102(l)(2), 21 C.F.R. part 56; 42 U.S.C. Sect. 241(d); 5 U.S.C. Sect. 552a; 44 U.S.C. Sect. 3501 et seq.

***
https://www.cdc.gov/fungal/diseases/ringworm/health-professionals.html

†††
https://www.accessdata.fda.gov/drugsatfda_docs/label/2014/018827s046lbl.pdf

§§§
https://www.aafp.org/pubs/afp/collections/choosing-wisely/471.html
